# Author Correction: The interferon stimulated gene-encoded protein HELZ2 inhibits human LINE-1 retrotransposition and LINE-1 RNA-mediated type I interferon induction

**DOI:** 10.1038/s41467-023-36226-4

**Published:** 2023-01-30

**Authors:** Ahmad Luqman-Fatah, Yuzo Watanabe, Kazuko Uno, Fuyuki Ishikawa, John V. Moran, Tomoichiro Miyoshi

**Affiliations:** 1 Department of Gene Mechanisms, Graduate School of Biostudies, Kyoto University, Kyoto, 606-8501 Japan; 2 Radiation Biology Center, Graduate School of Biostudies, Kyoto University, Kyoto, 606-8501 Japan; 3 Proteomics Facility, Graduate School of Biostudies, Kyoto University, Kyoto, 606-8501 Japan; 4 Division of Basic Research, Louis Pasteur Center for Medical Research, Kyoto, 606-8225 Japan; 5 Department of Human Genetics, University of Michigan Medical School, Ann Arbor, MI 48109-5618 USA; 6 Department of Internal Medicine, University of Michigan Medical School, Ann Arbor, MI 48109-5618 USA; 7 Laboratory for Retrotransposon Dynamics, RIKEN Center for Integrative Medical Sciences, Yokohama, 230-0045 Japan

**Keywords:** RNA metabolism, Genomic instability, Innate immunity

Correction to: *Nature Communications* 10.1038/s41467-022-35757-6, published online 13 January 2023

In this article the affiliation ‘Laboratory for Retrotransposon Dynamics, RIKEN Center for Integrative Medical Sciences, Yokohama 230-0045, Japan’ for Tomoichiro Miyoshi was missing. The original article has been corrected.

