# Supplementary material for: The interferon stimulated gene-encoded protein HELZ2 inhibits human LINE-1 retrotransposition and LINE-1 RNA-mediated type I interferon induction
Source: Nat Commun. 2023 Jan 13;14:203. doi: 10.1038/s41467-022-35757-6 (PMC9839780; doi:10.1038/s41467-022-35757-6)
Supplement: Supplementary file 1 — Supplementary Information [file 41467_2022_35757_MOESM1_ESM.pdf]

Supplementary Information for

The interferon stimulated gene-encoded protein HELZ2 inhibits human LINE-1 retrotransposition and LINE-1 RNA-mediated type I interferon induction

Luqman-Fatah A. *et al*.

Supplementary Figures 1-6

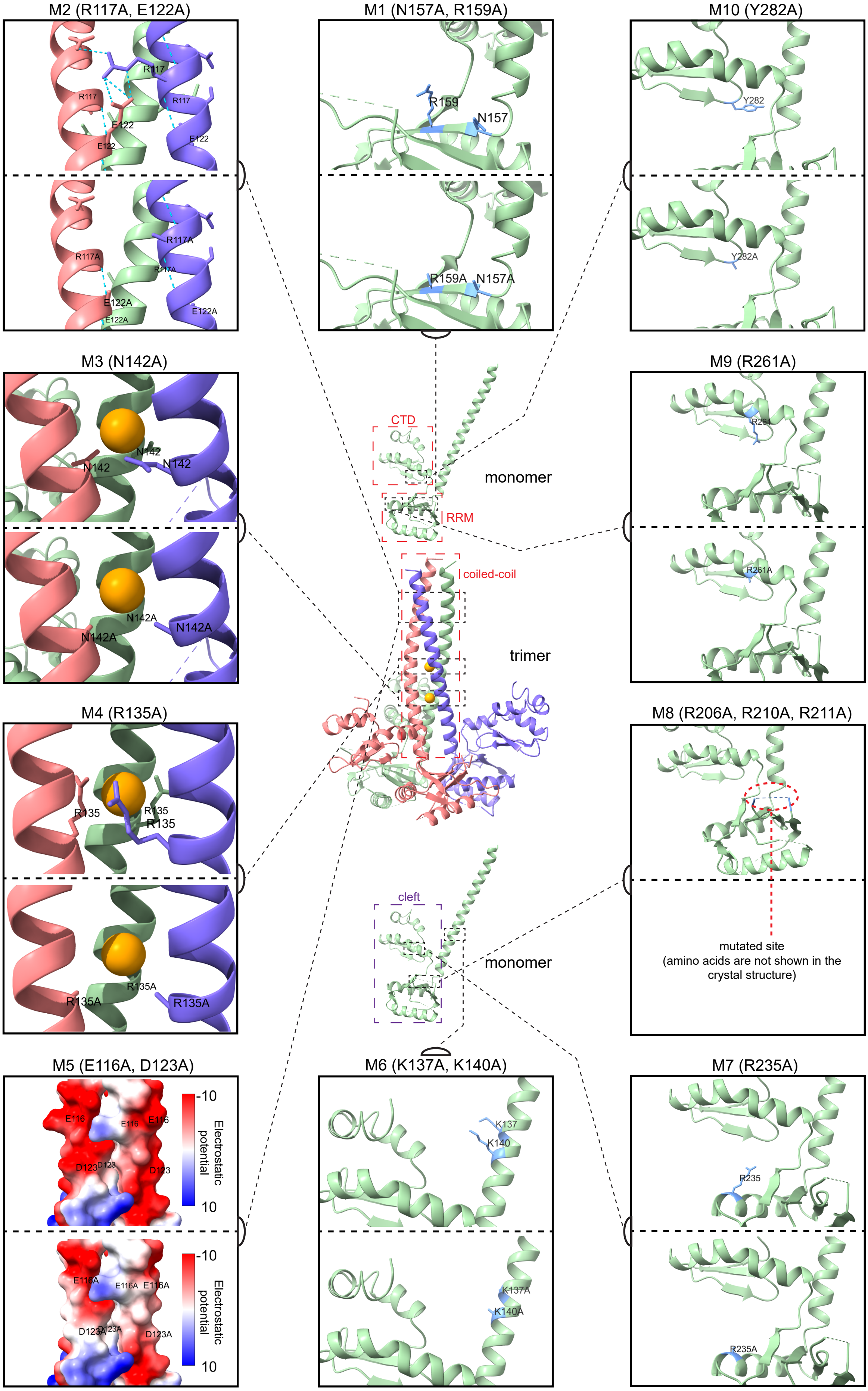

M2 (R117A, E122A)
R117
R117
R117
E122
E122
E122
R117A
R117A
R117A
E122A
E122A
E122A
M1 (N157A, R159A)
R159
N157
R159A
N157A
M10 (Y282A)
Y282
Y282A
M3 (N142A)
N142
N142
N142
N142A
N142A
N142A
M9 (R261A)
R261
R261A
CTD
RRM
monomer
coiled-coil
trimer
M4 (R135A)
R135
R135
R135
R135A
R135A
R135A
M8 (R206A, R210A, R211A)
mutated site
(amino acids are not shown in the crystal structure)
cleft
monomer
M5 (E116A, D123A)
E116
E116
E116
D123
D123
D123
-10
10
Electrostatic potential
E116A
E116A
E116A
D123A
D123A
D123A
-10
10
Electrostatic potential
M6 (K137A, K140A)
K137
K140
K137A
K140A
M7 (R235A)
R235
R235A

Supplementary Figure. 1 (supporting Fig. 1a and Supplementary Figs. 2a and 2b): Crystal structure of L1 ORF1p mutants.

Center: Crystal structure of the ORF1p trimer (middle) and monomer (top and bottom). Shown is the crystal structure assembly of the ORF1p trimer from amino acid residues 107 to 323 in the "lifted" conformation (Protein Data Bank ID: 2ykp); each monomer is annotated with distinct colors (green, purple, and red colors). Two chloride ion residues (orange spheres) are shown in the predicted position inside the coiled-coil domain (red-dotted box, top of the trimer). Each monomer forms a flexible cleft (indicated in bottom monomer: purple-dotted box) made up of an RNA recognition motif (top monomer: RRM, bottom of the cleft, red-dotted box) and a C-terminal domain (top monomer: CTD, top of the cleft, red-dotted box) to bind RNA. Relative positions of the mutated amino acids are indicated in black-dotted boxes connected with black-dotted lines to the respective enlarged images of the mutated sites. **Periphery:** Mutated sites of the ORF1p mutants in this study. Based on the number from lowest (*e.g.,* M1 and M2) to highest (*e.g.*, M10), the ORF1p mutants were arranged in a counterclockwise direction beginning from the top (middle), where each of the mutant is enclosed in black boxes with the respective annotation noted at the top. Amino acids corresponding to the WT and alanine missense mutations are indicated within each box, where the WT (upper) and the mutants (lower) are separated by black-dotted lines. M1, M6, M7, M9 and M10: mutated amino acids and side chains are indicated in blue. M2: blue-dotted lines indicate hydrogen bonds formed, including between R117 and E122 side chains (different monomers) to stabilize the trimer. M3 and M4: depicted are the predicted side chains thought to stabilize the chloride ions. M5: a relative electrostatic potential map of the ORF1p trimers surface, the mutated site was suggested to be a potential recruitment site of host factors. Red indicates low positive electrostatic potential (high acidity) and blue indicates high positive electrostatic potential (high basicity). M8: the mutated site is shown in the red-dotted circle.

a

Number	M1	M2	M3	M4	M5	M6	M7	M8	M9	M10
Mutational Sites	N157A, R159A	R117A, E122A	N142A	R135A	E116A, D123A	K137A, K140A	R235A	R206A, R210A, R211A	R261A	Y282A
Putative functions	Conserved site, abolished ORF1p foci	RhxxhE motif (trimerization)	Chloride ion mediator (trimerization)	Chloride ion mediator (trimerization)	Putative protein binding site, does not affect RNA binding	Decrease RNA binding (coiled-coil)	Decrease RNA binding (RRM)	Loss of RNA binding ability (RRM)	Decrease RNA binding and chaperone activity (CTD)	Decrease chaperone activity (CTD)
References	Goodier, JL. *et al* ., 2007	Kammerer, RA. et al., 2005	Khazina, E. *et al.,* 2011	Khazina, E. *et al.,* 2011	Khazina, E. *et al.,* 2011	Khazina, E. *et al.,* 2011	Doucet, AJ. *et al.* , 2010	Khazina, E. *et al.,* 2011	Doucet, AJ. *et al.* , 2010	Doucet, AJ. *et al.* , 2010

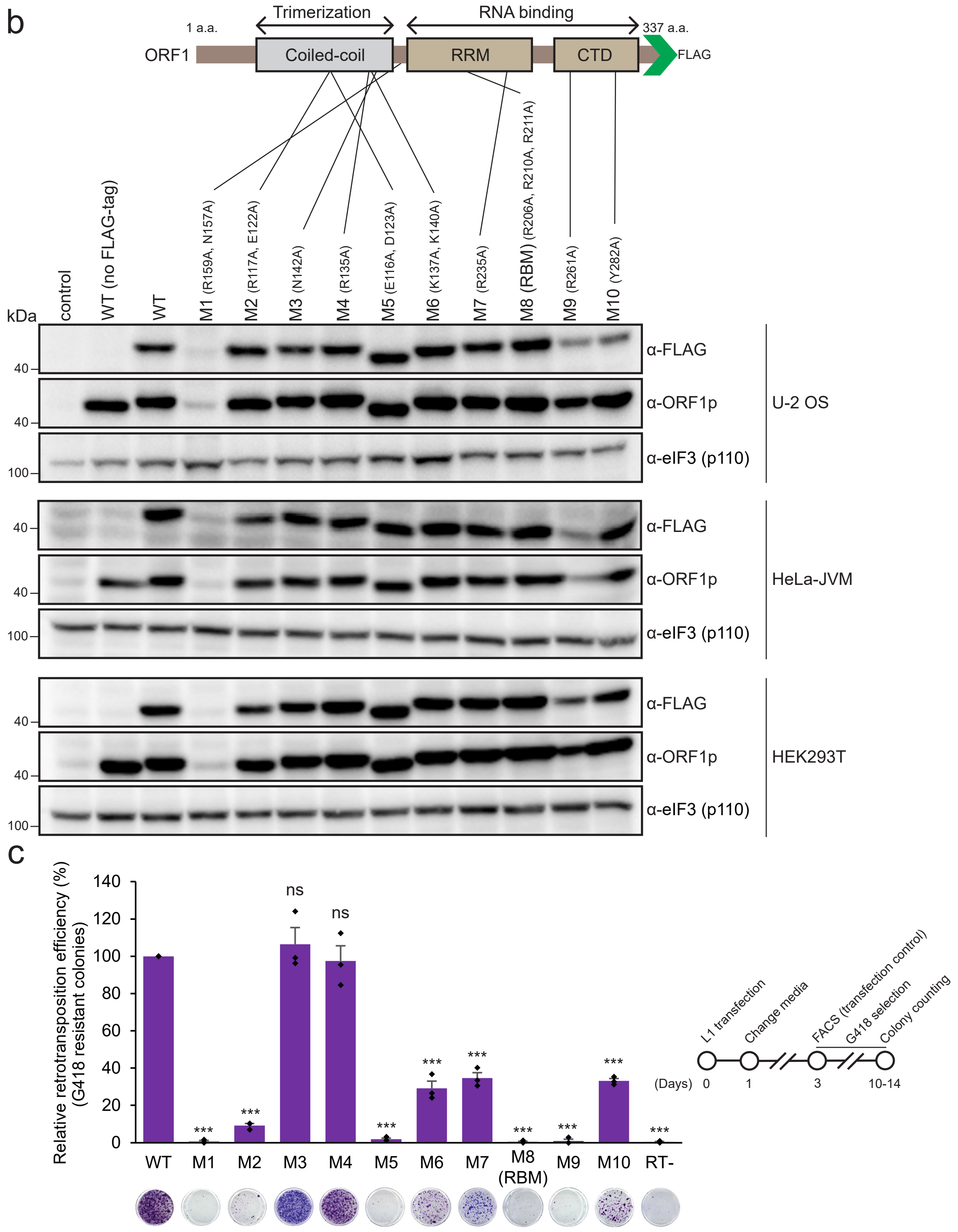

Supplementary Figure. 2 (supporting Figs. 1a, 1b, and 1d): L1 ORF1p mutational analyses.

(a) ORF1p mutants generated in this study*.* Ten alanine missense ORF1p-FLAG mutants (M1 to M10) were tested in various assays. Row 1, mutant number. Row 2, alanine mutations; commas denote double (*i.e.*, M1, M2, M5, M6) or triple (*i.e.*, M8) mutants. Row 3, putative functional domains affected by the alanine mutations. Row 4, references to previous studies implicating the mutations in L1 biology. Some of the mutants were designed based upon the ORF1p crystal structure. **(b)** Schematic representation of ORF1p functional domains containing the mutations noted in panel (a)***.*** Top, relative positions of the respective mutated amino acids. Bottom, western blots to test whether the relative mutations are expressed in U-2 OS, HeLa-JVM, or HEK293T cells. The cells were transfected with: pJM101/L1.3FLAG (WT); pALAF001 (M1); pALAF002 (M2); pALAF003 (M3); pALAF004 (M4); pALAF005 (M5); pALAF006 (M6); pALAF007 (M7); pALAF008 (M8); pALAF009 (M9); or pALAF010 (M10). U-2 OS, HeLa-JVM, or HEK293T cells were collected on day 5, day 9, or day 4 post-transfection, respectively, which were determined to be the optimal days to observe ORF1p steady state levels in the respective cell lines. An anti-FLAG antibody was used to detect ORF1p-FLAG. An anti-ORF1p antibody was used to ensure that the ORF1p-FLAG signals correspond to the ORF1p levels. The eIF3 protein (p110) served as a loading control. **(c)** L1 retrotransposition efficiencies***.*** HeLa-JVM cells were co-transfected with the plasmids used in panel (b) and a phrGFP-C plasmid to normalize for transfection efficiencies and subjected to *mneoI*-based retrotransposition assays (inset, timeline of the assay). X-axis, mutant name, and representative results from the assay; a missense mutation in the ORF2p RT domain (RT-) served as a negative control. Y-axis, the percentage of normalized G418-resistant foci compared to the WT (pJM101/L1.3FLAG) control. Pairwise comparisons relative to the WT control: p = 1.8 x 10^{-12}*** (M1); 7.6 x 10^{-12}*** (M2); 0.56^{ns} (M3); 0.67^{ns} (M4); 2.1 x 10^{-12}*** (M5); 5.7 x 10^{-10}*** (M6); 1.4 x 10^{-9}*** (M7); 2.1 x 10^{-12}*** (M8); 2.0 x 10^{-12}*** (M9); 1.3 x 10^{-9}*** (M10); 2.0 x 10^{-12}*** (RT-). Values represent the mean ± SEM of three independent biological replicates. The *p*-values were calculated using a one-way ANOVA followed by Bonferroni-

Holm post-hoc tests: ns: not significant; *** $p<0.001$. The relative retrotransposition efficiencies and the representative results reported in Fig. 1d were taken from this retrotransposition assay (WT and M8 [RBM]).

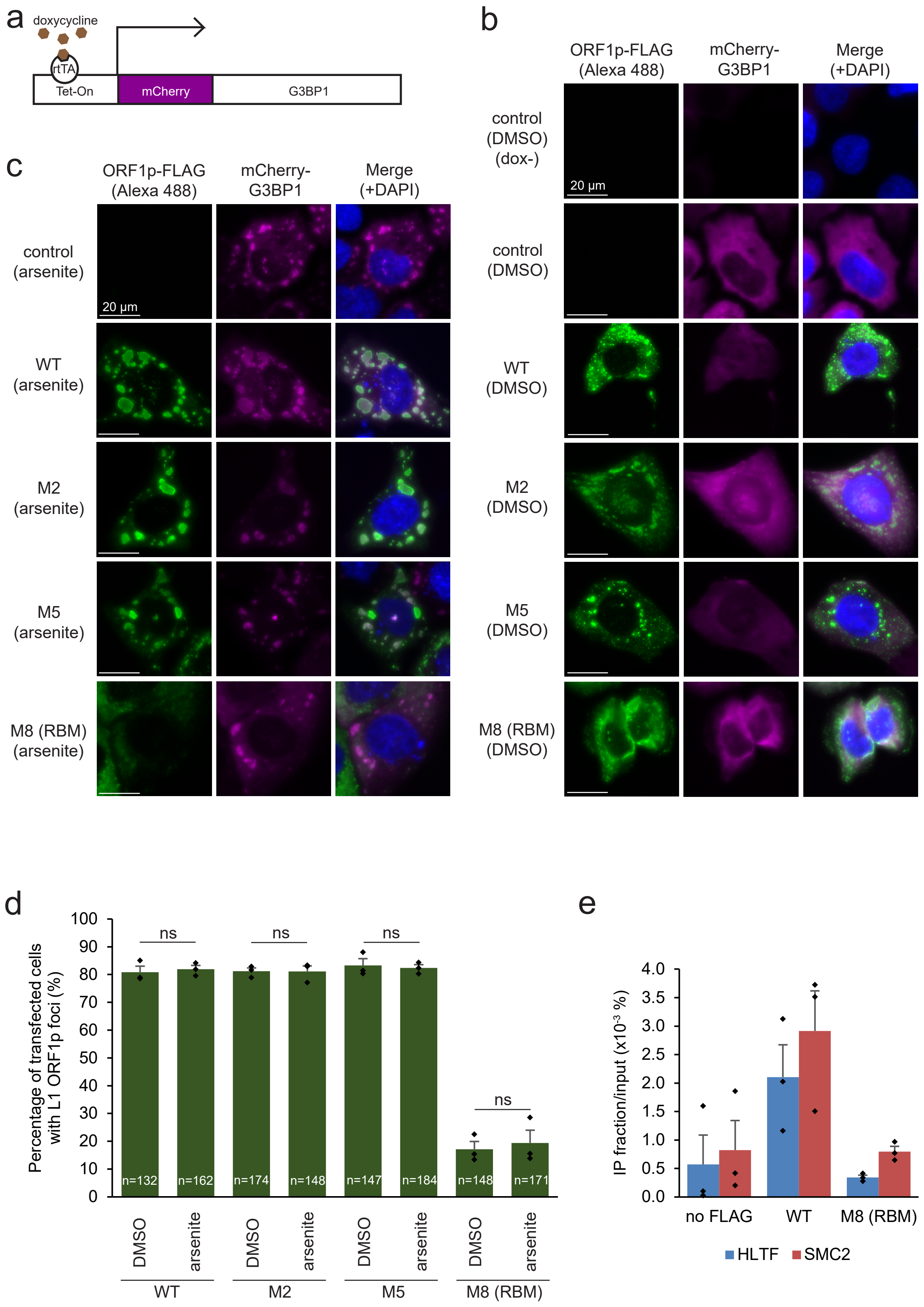

a
doxycycline
rtTA
Tet-On
mCherry
G3BP1
b
ORF1p-FLAG (Alexa 488)
mCherry-G3BP1
Merge (+DAPI)
control (DMSO) (dox-)
20 µm
control (DMSO)
WT (DMSO)
M2 (DMSO)
M5 (DMSO)
M8 (RBM) (DMSO)
c
ORF1p-FLAG (Alexa 488)
mCherry-G3BP1
Merge (+DAPI)
control (arsenite)
20 µm
WT (arsenite)
M2 (arsenite)
M5 (arsenite)
M8 (RBM) (arsenite)
d
Percentage of transfected cells with L1 ORF1p foci (%)
ns
n=132
n=162
n=174
n=148
n=147
n=184
n=148
n=171
DMSO
arsenite
WT
M2
M5
M8 (RBM)
e
IP fraction/input (x10^{-3} %)
no FLAG
WT
M8 (RBM)
HLTF
SMC2

Supplementary Figure. 3 (supporting Figs. 1e, 1f, and 1g): L1 cytoplasmic foci formation with the ORF1p mutants.

(a) Schematic of the doxycycline inducible mCherry-G3BP1 expression plasmid. An mCherry-G3BP1 fusion protein only will be expressed in U-2 OS cells when doxycycline binds to the reverse tetracycline-controlled *trans*-activator protein (rtTA) and rtTA subsequently binds to the Tet-On promoter to activate mCherry-G3BP1 transcription. **(b and c)** Representative immunofluorescence images of WT, M2, M5, and M8 (RBM) ORF1p localization in the absence (b) or presence (c) of arsenite. U-2 OS cells containing the inducible mCherry-G3BP1 expression cassette were transfected with pCEP4 (control), pJM101/L1.3FLAG (WT), pALAF002 (M2), pALAF005 (M5), or pALAF008 (M8). Two days post-transfection, the cells were treated with DMSO or 0.5 mM sodium arsenite for 1 hour prior to fixation. A mouse primary anti-FLAG antibody and secondary anti-mouse-Alexa Fluor 488 fluorescent dye-conjugated antibodies were used to visualize ORF1p. Cells not treated with doxycycline (dox-) were included as a control in panel (b). White bars, 20 µm. **(d)** Quantification of ORF1p-FLAG cytoplasmic foci in U-2 OS cells transfected with WT, M2, M5, or M8 (RBM) ORF1p L1 expression constructs. X-axis, construct name and whether the cells were treated with vehicle (DMSO) or arsenite. Y-axis, the percentage of transfected cells exhibiting ORF1p-FLAG cytoplasmic foci. The numbers (n) within the green rectangles indicate the number of cells analyzed in the experiment. The percentage of transfected cells with L1 ORF1p foci data in Fig. 1f were taken from the WT (DMSO) and M8 (RBM) (DMSO) samples. Pairwise comparisons between DMSO and arsenite-treated cells: $p = 1.00^{ns}$ (WT); 1.00^{ns} (M2); 1.00^{ns} (M5); 1.00^{ns} (M8 [RBM]). **(e)** RNA-immunoprecipitation (RNA-IP) reveals a general RNA binding defect in the ORF1p-FLAG R206A/R210A/R211A mutant (M8 [RBM]). HeLa-JVM cells were transfected with either pJM101/L1.3 (no FLAG), pJM101/L1.3FLAG (WT ORF1p-FLAG), or the ORF1p-FLAG R206A/R210A/R211A mutant pALAF008 (M8 [RBM]). An anti-FLAG antibody was used to immunoprecipitate ORF1p-FLAG; reverse transcription-quantitative PCR (RT-qPCR) using primer sets (HLTF and SMC2, respectively) that amplify RNAs derived from genes previously reported to be enriched in ORF1p RNA-IP experiments. X-axis,

construct name. Y-axis, the enrichment of RNA levels between the IP and input fractions. Blue rectangles, relative levels of HLTF RNA. Red rectangles, relative levels SMC2 RNA. Values represent the mean ± SEM of three independent biological replicates. The *p*-values were calculated using a one-way ANOVA followed by Bonferroni-Holm post-hoc tests. ns: not significant.

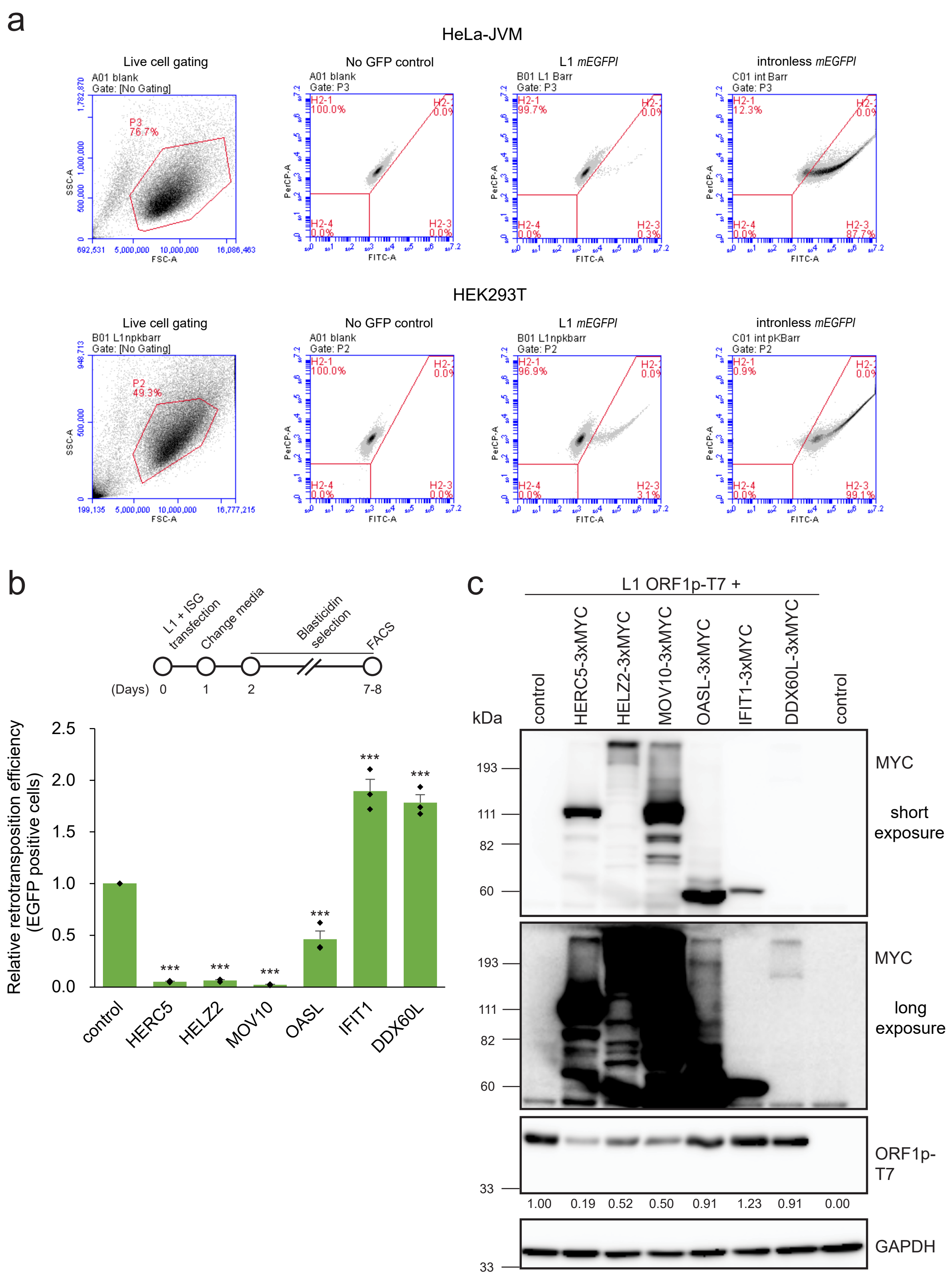
a
HeLa-JVM
Live cell gating
No GFP control
L1 mEGFPI
intronless mEGFPI
HEK293T
b
L1 + ISG transfection
Change media
Blasticidin selection
FACS
(Days) 0 1 2 7-8
Relative retrotransposition efficiency (EGFP positive cells)
control
HERC5
HELZ2
MOV10
OASL
IFIT1
DDX60L
c
L1 ORF1p-T7 +
control
HERC5-3xMYC
HELZ2-3xMYC
MOV10-3xMYC
OASL-3xMYC
IFIT1-3xMYC
DDX60L-3xMYC
control
kDa
MYC
short exposure
MYC
long exposure
ORF1p-T7
1.00 0.19 0.52 0.50 0.91 1.23 0.91 0.00
GAPDH

Supplementary Figure. 4 (supporting Figs. 4a, 4b, 5b, 5c and 5e; Supplementary Figs. 4b, 5e, 5f, 5g, and 5h): Functional analysis of the ISG proteins in HEK293T cells.

(a) Representative flow cytometry plots of L1 mEGFPI retrotransposition assays in HeLa-JVM (top) and HEK293T (bottom). Thirty thousand live cells were gated in the hexagon-shaped box (*i.e.*, P3 (HeLa-JVM) and P2 (HEK293T)). X-axis, forward scatter-area (FSC-A) channel. Y-axis, side scatter-area (SSC-A) channel (leftmost plot, live cell gating). EGFP+ cells were detected using Fluorescein isothiocyanate-area (FITC-A) channel (x-axis) with peridinin chlorophyll protein complex-area (PerCP-A) (y-axis) channel as a control. A threshold line was set accordingly based on the negative control (middle left plot, No GFP control). The H2-3 fraction indicates percentage of EGFP+ cells. Shown are representative plots of an L1 containing the EGFP reporter cassette (*mEGFPI*) (middle right plot) and an intronless EGFP reporter cassette, which serves as a transfection control (rightmost plot). **(b)** Overexpression of HERC5, HELZ2, and OASL inhibit L1 retrotransposition in HEK293T*.* Top: the timeline of the assay. HEK293T cells we co-transfected with cep99-gfp-L1.3 (which has the *mEGFPI* retrotransposition indicator cassette) and either pCEP4 (control) or the following individual ISG protein expression plasmids containing three copies of a MYC epitope tag (3xMYC) at their respective carboxyl termini: pALAF015 (HELZ2); pALAF016 (IFIT1); pALAF021 (DDX60L); pALAF022 (OASL); pALAF023 (HERC5); or pALAF024 (MOV10). EGFP-positive cells transfected with cep99-gfp-L1.3 were counted using flow cytometry and normalized to the number of EGFP-positive cells in the transfection control (*i.e.*, cells independently transfected with the cep99-gfp-L1.3RT(-) intronless plasmid and each of the above listed plasmids). X-axis, name of constructs co-transfected with cep99-gfp-L1.3. Y-axis, relative percentage of EGFP-positive cells relative to the cep99-gfp-L1.3 + pCEP4 control. Pairwise comparisons relative to the control: $p = 4.8 \times 10^{-7}$*** (HERC5); 4.6×10^{-7}*** (HELZ2); 6.1×10^{-7}*** (MOV10); 3.9×10^{-5}*** (OASL); 6.2×10^{-7}*** (IFIT1); 1.5×10^{-6}*** (DDX60L). Values represent the mean ± SEM from three independent biological replicates. The *p*-values were calculated using a one-way ANOVA followed by Bonferroni-Holm post-hoc tests (*** $p<0.001$). **(c)** Western blot detection of ORF1p in HEK293T cells co-transfected with ISG-expressing plasmids. HEK293T

cells were co-transfected with pTMF3 (L1 containing T7 epitope-tagged ORF1p) and either pCMV-3Tag-8-Barr (control) or the individual ISG-expressing plasmids used in panel (b). The relative band intensities of ORF1p-T7 are indicated under the ORF1p-T7 blot. They were calculated using ImageJ software and are normalized to the respective GAPDH band intensities. An anti-MYC antibody was used to detect the ISG proteins, and the western blot was shown as the short (top) and long exposure (bottom) images. An anti-T7 antibody was used to detect WT ORF1p-T7. GAPDH served as a sample processing control. Molecular weight markers (kDa) are indicated at the left of the blots.

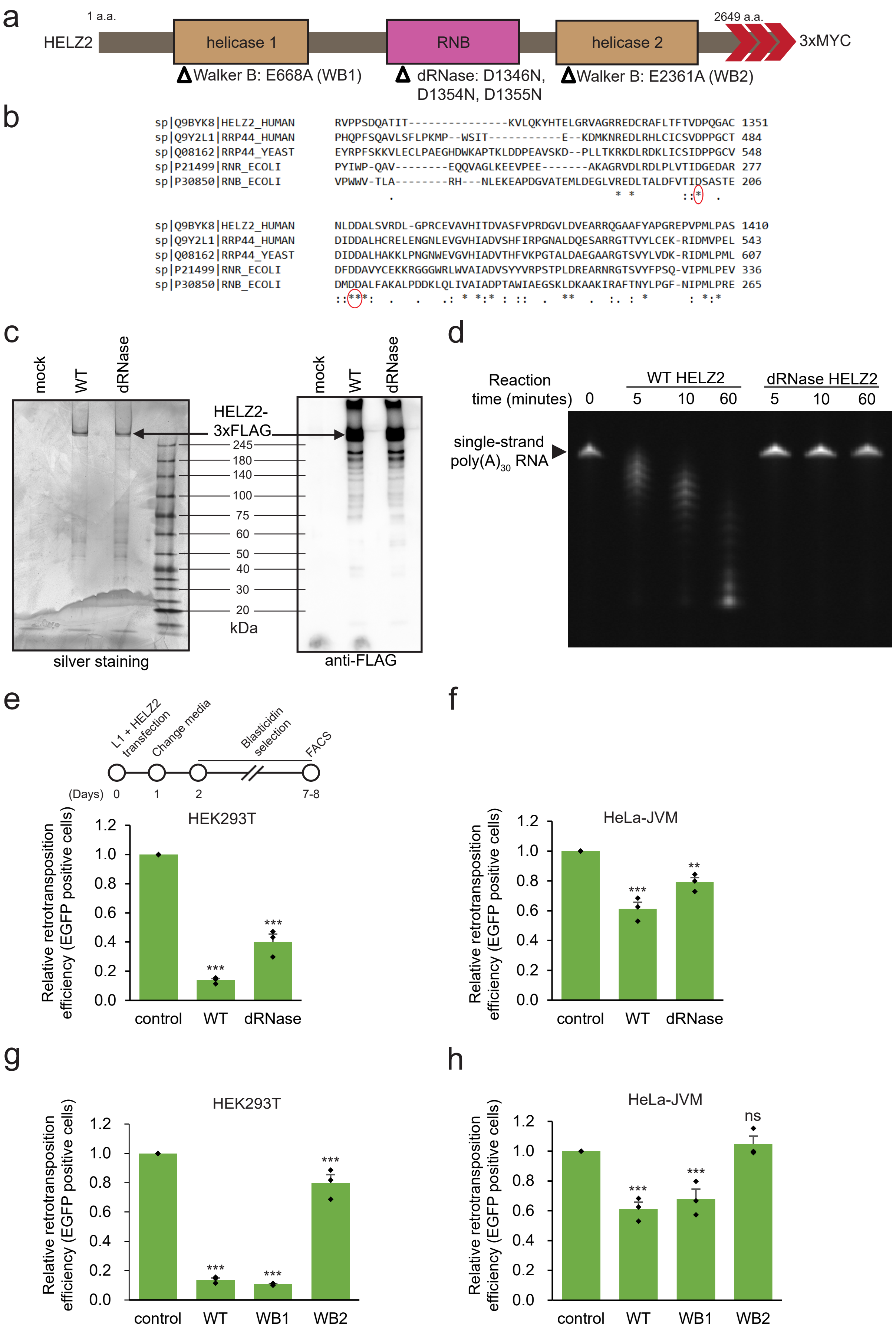
a
HELZ2
1 a.a.
helicase 1
RNB
helicase 2
2649 a.a.
3xMYC
Walker B: E668A (WB1)
dRNase: D1346N, D1354N, D1355N
Walker B: E2361A (WB2)
b
sp|Q9BYK8|HELZ2_HUMAN RVPPSDQATIT---------------KVLQKYHTELGRVAGRREDCRAFLTFTVDPQGAC 1351
sp|Q9Y2L1|RRP44_HUMAN PHQPFSQAVLSFLPKMP--WSIT-----------E--KDMKNREDLRHLCICSVDPPGCT 484
sp|Q08162|RRP44_YEAST EYRPFSKKVLECLPAEGHDWKAPTKLDDPEAVSKD--PLLTKRKDLRDKLICSIDPPGCV 548
sp|P21499|RNR_ECOLI PYIWP-QAV--------EQQVAGLKEEVPEE-------AKAGRVDLRDLPLVTIDGEDAR 277
sp|P30850|RNB_ECOLI VPWWV-TLA--------RH---NLEKEAPDGVATEMLDEGLVREDLTALDFVTIDSASTE 206
sp|Q9BYK8|HELZ2_HUMAN NLDDALSVRDL-GPRCEVAVHITDVASFVPRDGVLDVEARRQGAAFYAPGREPVPMLPAS 1410
sp|Q9Y2L1|RRP44_HUMAN DIDDALHCRELENGNLEVGVHIADVSHFIRPGNALDQESARRGTTVYLCEK-RIDMVPEL 543
sp|Q08162|RRP44_YEAST DIDDALHAKKLPNGNWEVGVHIADVTHFVKPGTALDAEGAARGTSVYLVDK-RIDMLPML 607
sp|P21499|RNR_ECOLI DFDDAVYCEKKRGGGWRLWVAIADVSYYVRPSTPLDREARNRGTSVYFPSQ-VIPMLPEV 336
sp|P30850|RNB_ECOLI DMDDALFAKALPDDKLQLIVAIADPTAWIAEGSKLDKAAKIRAFTNYLPGF-NIPMLPRE 265
c
mock
WT
dRNase
HELZ2-3xFLAG
245
180
140
100
75
60
50
40
30
20
kDa
silver staining
anti-FLAG
d
Reaction time (minutes)
0
WT HELZ2
5
10
60
dRNase HELZ2
5
10
60
single-strand poly(A)30 RNA
e
L1 + HELZ2 transfection
Change media
Blasticidin selection
FACS
(Days) 0
1
2
7-8
HEK293T
Relative retrotransposition efficiency (EGFP positive cells)
1.2
1.0
0.8
0.6
0.4
0.2
0.0
***
***
control
WT
dRNase
f
HeLa-JVM
Relative retrotransposition efficiency (EGFP positive cells)
1.2
1.0
0.8
0.6
0.4
0.2
0.0
***
**
control
WT
dRNase
g
HEK293T
Relative retrotransposition efficiency (EGFP positive cells)
1.2
1.0
0.8
0.6
0.4
0.2
0.0
***
***
***
control
WT
WB1
WB2
h
HeLa-JVM
Relative retrotransposition efficiency (EGFP positive cells)
1.2
1.0
0.8
0.6
0.4
0.2
0.0
***
***
ns
control
WT
WB1
WB2

Supplementary Figure. 5 (supporting Figs. 5b, 5c, and 5d): Functional analyses of the HELZ2 RNB and helicase domains.

(a) Schematic of mutations in the HELZ2 RNB domain*.* The HELZ2 protein contains two putative helicase domains (helicase 1 and helicase 2), which surround a putative RNB exonuclease domain. Open triangles, positions of the missense mutations in conserved amino acids within the Walker B boxes in the helicase 1 and helicase 2 domains (E668A [WB1] and E2361A [WB2], respectively). The RNB mutant contains three missense mutations (D1346N/D1354N/D1355N [dRNase]). Three red arrowheads, relative positions of the 3xMYC carboxyl-terminal epitope tags. **(b)** Identification of conserved amino acids in the RNB domain*.* Multiple sequence alignments of the following RNB-containing proteins: *Homo sapiens* exosome complex exonuclease Rrp44 (RRP44_HUMAN) and HELZ2 (HELZ2_HUMAN); *Saccharomyces cerevisiae* exosome complex exonuclease Rrp44 (RRP44_YEAST); and *Escherichia coli* RNase R (RNR_ECOLI) and Exoribonuclease 2 (RNB_ECOLI). Red circles, amino acids mutated in the D1346N/D1354N/D1355N (dRNase) triple mutant. **(c)** Recombinant WT and mutant dRNase HELZ2 protein purification. The hHELZ2_3xFLAG (pALAF071) and hHELZ2_3xFLAG_dRNase (pALAF073) plasmid constructs were transfected in HEK293T cells and the corresponding proteins were purified using anti-FLAG antibody, subjected to SDS-PAGE, and visualized by silver staining (left) and western blot using an anti-FLAG antibody (right). The pCEP4-transfected cells served as a negative control (mock). The arrow indicates the position of the HELZ2-3xFLAG protein. **(d)** HELZ2 has a 3' to 5' RNase activity. The WT HELZ2-3xFLAG and dRNase HELZ2-3xFLAG proteins in panel (c) were incubated with a single-strand poly(A)$_{30}$ RNA oligonucleotide labeled with IRDye800 at its 5' end (poly[rA$_{30}$]) for 0, 5, 10, and 60 minutes at 37˚C. Reactions containing single-strand poly(A)$_{30}$ RNA that lacked the recombinant protein served as a negative control (leftmost lane). The single-stranded (ss) RNAs were separated on a polyacrylamide/urea gel with 1x TBE buffer. **(e)** L1 retrotransposition efficiency in the presence of the D1346N/D1354N/D1355N (dRNase) mutant in HEK293T cells. Top: the timeline for the retrotransposition assays shown in panels (e), (f), (g), and (h). HEK293T cells were co-transfected with cepB-gfp-L1.3 (*mEGFPI*)

and either pCMV-3Tag-8-Barr (control), pALAF015 (WT), or pALAF030 (dRNase). The retrotransposition efficiency was normalized to the transfection efficiency control (*i.e.*, cells co-transfected with cepB-gfp-L1.3RT(-) intronless and either pCMV-3Tag-8-Barr (control), pALAF015 (WT), or pALAF030 (dRNase)). X-axis, name of the plasmid co-transfected with cepB-gfp-L1.3 (*mEGFPI*). Y-axis, relative retrotransposition efficiency relative to the cepB-gfp-L1.3 (*mEGFPI*) + pCMV-3Tag-8-Barr control. Pairwise comparisons relative to the cepB-gfp-L1.3 (*mEGFPI*) + pCMV-3Tag-8-Barr control: p = 9.4 x 10^{-10}*** (WT HELZ2), 4.1 x 10^{-8}*** (dRNase). **(f)** L1 retrotransposition efficiency in the presence of the D1346N/D1354N/D1355N (dRNase) mutant in HeLa-JVM cells. Experiments were conducted as summarized in panel (e). Pairwise comparisons relative to the control: p = 9.5 x 10^{-5}*** (WT); 0.0073** (dRNase). **(g)** The effects of mutations in the Walker B box on L1 retrotransposition in HEK293T cells. HEK293T cells were co-transfected with cepB-gfp-L1.3, which contains an *mEGFPI* retrotransposition indicator cassette, and either pCMV-3Tag-8-Barr (control), pALAF015 (WT HELZ2), or one of the following HELZ2 expression plasmids that contain a mutation(s) in the Walker B box (*i.e.*, pALAF028 [WB1] or pALAF029 [WB2]). Cells co-transfected with cepB-gfp-L1.3RT(-) intronless and either pCMV-3Tag-8-Barr, pALAF015 (WT HELZ2), or a mutant HELZ2 plasmid served as transfection, normalization, and toxicity controls. Retrotransposition efficiencies were calculated as described in panel (e). Pairwise comparisons relative to the cepB-gfp-L1.3 (mEGFPI) + pCMV-3Tag-8-Barr control: p = 9.4 x 10^{-10}*** (WT); 8.4 x 10^{-10}*** (WB1); and 8.7 x 10^{-4}*** (WB2). **(h)** The effect of mutations in the Walker B box on L1 retrotransposition in HeLa-JVM cells. HeLa-JVM cells were co-transfected as in panel (g). Retrotransposition efficiencies were calculated as described in panel (e). Pairwise comparisons relative to the L1.3 + pCMV-3Tag-8-Barr control: p = 9.5 x 10^{-5}*** (WT); 0.0004*** (WB1); and 0.43^{ns} (WB2). Values represent the mean ± SEM of three independent biological replicates. The p-values were calculated using a one-way ANOVA followed by Bonferroni-Holm post-hoc tests. ns: not significant; *** p<0.001; ** p<0.01.

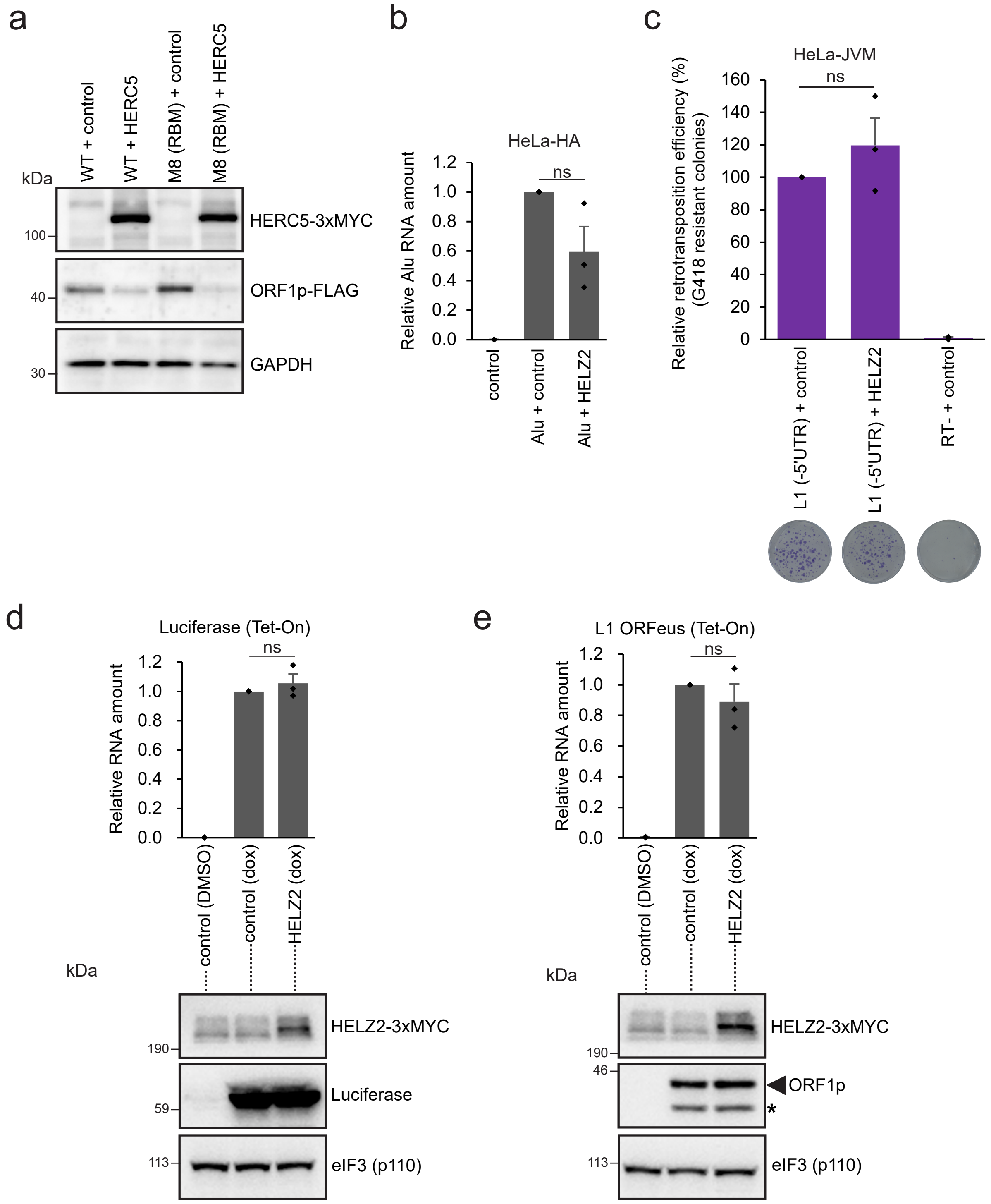

a
kDa
WT + control
WT + HERC5
M8 (RBM) + control
M8 (RBM) + HERC5
HERC5-3xMYC
100
ORF1p-FLAG
40
GAPDH
30
b
HeLa-HA
Relative Alu RNA amount
1.2
1.0
0.8
0.6
0.4
0.2
0.0
ns
control
Alu + control
Alu + HELZ2
c
HeLa-JVM
Relative retrotransposition efficiency (%)
(G418 resistant colonies)
160
140
120
100
80
60
40
20
0
ns
L1 (-5'UTR) + control
L1 (-5'UTR) + HELZ2
RT- + control
d
Luciferase (Tet-On)
Relative RNA amount
1.2
1.0
0.8
0.6
0.4
0.2
0.0
ns
control (DMSO)
control (dox)
HELZ2 (dox)
kDa
HELZ2-3xMYC
190
Luciferase
59
eIF3 (p110)
113
e
L1 ORFeus (Tet-On)
Relative RNA amount
1.2
1.0
0.8
0.6
0.4
0.2
0.0
ns
control (DMSO)
control (dox)
HELZ2 (dox)
kDa
HELZ2-3xMYC
190
46
ORF1p
*
eIF3 (p110)
113

Supplementary Figure. 6 (supporting Figs. 6b, 6c, 6d, 6e, and 6f): The L1 5'UTR is required for the HELZ2-mediated reduction in L1 RNA steady state levels.

(a) HERC5 expression reduces steady state levels of L1 ORF1p independently of ORF1p RNA-binding. HeLa-JVM cells were co-transfected with pJM101/L1.3FLAG (WT ORF1p-FLAG) or the pALAF008 (M8 [RBM] ORF1p-FLAG) mutant expression plasmid and either pCMV-3Tag-8-Barr (control) or pALAF023 (HERC5). ORF1p-FLAG and HERC5 protein levels were detected by western blot using anti-MYC and anti-FLAG (Sigma Aldrich, F7425) antibodies, respectively. GAPDH served as a sample processing control. **(b)** HELZ2 modestly reduces Alu RNA levels***.*** HeLa-HA cells were transfected with only pCMV-3Tag-8-barr (control), or co-transfected with Alu-*neo*^{Tet} (Alu) and either pCMV-3Tag-8-barr (control) or pALAF015 (HELZ2). X-axis, constructs name. Y-axis, the relative amount of Alu RNA (primer set: *mneoI* [Alu or L1]). Pairwise comparison: p = 0.64^{ns}. **(c)** The L1 5'UTR is required for HELZ2-mediated reduction of L1 retrotransposition***.*** HeLa-JVM cells were co-transfected with L1 (Δ5'UTR) and either pCMV-3Tag-8-barr (control) or pALAF015 (HELZ2). Another set of HeLa-JVM cells were co-transfected with pCMV-3Tag-8-barr (control) or pALAF015 (HELZ2) and a phrGFP-C plasmid to normalize for transfection efficiencies and subjected to *mneoI*-based retrotransposition assays. X-axis, constructs name and representative results from the assay; a missense mutation in the ORF2p RT domain (RT-) served as a negative control. Y-axis, the percentage of normalized G418-resistant foci compared to the WT (pJM101/L1.3FLAG) control. Pairwise comparison: p = 0.2^{ns}. **(d & e)** The effect of HELZ2 on doxycycline inducible (Tet-On) luciferase (panel [d]) or human L1 ORFeus (panel [e]) expression*.* HeLa-JVM cells expressing inducible firefly luciferase (pSBtet-RN) or human L1 ORFeus (pDA093) were treated with vehicle (DMSO) or doxycycline (dox) and then transfected with either pCMV-3Tag-8-Barr (control) or pALAF015 (HELZ2). Cells were collected 48 hours post-transfection. Top: Luciferase and L1 levels were quantified using RT-qPCR (primer set: Luciferase and L1 [SV40], respectively) and normalized to *GAPDH* RNA levels (primer set: GAPDH). X-axis, construct name and whether cells were treated with vehicle (DMSO) or doxycycline (dox). Y-axis, RNA levels normalized to the inducible firefly

luciferase (pSBtet-RN) or human L1 ORFeus (pDA093) + pCMV-3Tag-8-Barr control. Bottom: western blot analyses. An anti-MYC antibody was used to detect HELZ2, an anti-luciferase antibody was used to detect luciferase, and an anti-ORF1p antibody was used to detect ORF1p. Black arrowhead (middle right blot), the expected ORF1p band; asterisk (middle right blot), unexpected lower molecular weight ORF1p band. The eIF3 subunit (p110) served as a loading control. Pairwise comparisons: p = 0.32^{ns} (Luciferase); and 0.28^{ns} (L1). Values represent the mean ± SEM of three independent biological replicates. The p-values were calculated using a one-way ANOVA followed by Bonferroni-Holm post-hoc tests; ns: not significant.
