# Supplementary material for: The interferon stimulated gene-encoded protein HELZ2 inhibits human LINE-1 retrotransposition and LINE-1 RNA-mediated type I interferon induction
Source: Nat Commun. 2023 Jan 13;14:203. doi: 10.1038/s41467-022-35757-6 (PMC9839780; doi:10.1038/s41467-022-35757-6)
Supplement: Supplementary file 2 — Description of Additional Supplementary Files [file 41467_2022_35757_MOESM2_ESM.pdf]

Description of additional supplementary files

File name : Supplementary Data 1-4

Supplementary Data 1. DAVID Gene Ontology analysis (Functional annotation set UP_KW (UniProt_KeyWord) biological process) of >0.5 cutoff of log2(abundance ratio WT vs M8[RBM])

Supplementary Data 2. Preranked Gene Set Enrichment Analysis of fold difference (log2 [abundance ratio of WT vs M8/RBM]), followed by Leading Edge Analysis

Supplementary Data 3. Bio-Plex analysis of cytokines and chemokines secreted in poly(I:C) or L1-transfected HEK293T cells

Supplementary Data 4. Interferome analysis showing ISGs with 5-fold increase/decrease by all IFNs from protein hits with >0.5 cutoff of log2(abundance ratio WT vs M8[RBM])
