# Supplementary material for: The interferon stimulated gene-encoded protein HELZ2 inhibits human LINE-1 retrotransposition and LINE-1 RNA-mediated type I interferon induction
Source: Nat Commun. 2023 Jan 13;14:203. doi: 10.1038/s41467-022-35757-6 (PMC9839780; doi:10.1038/s41467-022-35757-6)
Supplement: Supplementary file 4 — Reporting Summary [file 41467_2022_35757_MOESM4_ESM.pdf]

nature portfolio

Corresponding author(s): Dr. Tomoichiro Miyoshi

Last updated by author(s): Dec 15, 2022

Reporting Summary

Nature Portfolio wishes to improve the reproducibility of the work that we publish. This form provides structure for consistency and transparency in reporting. For further information on Nature Portfolio policies, see our Editorial Policies and the Editorial Policy Checklist.

Statistics

For all statistical analyses, confirm that the following items are present in the figure legend, table legend, main text, or Methods section.

n/a	Confirmed	
☐	☒	The exact sample size (*n*) for each experimental group/condition, given as a discrete number and unit of measurement
☐	☒	A statement on whether measurements were taken from distinct samples or whether the same sample was measured repeatedly
☐	☒	The statistical test(s) used AND whether they are one- or two-sided<br>*Only common tests should be described solely by name; describe more complex techniques in the Methods section.*
☒	☐	A description of all covariates tested
☐	☒	A description of any assumptions or corrections, such as tests of normality and adjustment for multiple comparisons
☐	☒	A full description of the statistical parameters including central tendency (e.g. means) or other basic estimates (e.g. regression coefficient) AND variation (e.g. standard deviation) or associated estimates of uncertainty (e.g. confidence intervals)
☐	☒	For null hypothesis testing, the test statistic (e.g. *F*, *t*, *r*) with confidence intervals, effect sizes, degrees of freedom and *P* value noted<br>*Give P values as exact values whenever suitable.*
☒	☐	For Bayesian analysis, information on the choice of priors and Markov chain Monte Carlo settings
☒	☐	For hierarchical and complex designs, identification of the appropriate level for tests and full reporting of outcomes
☒	☐	Estimates of effect sizes (e.g. Cohen's *d*, Pearson's *r*), indicating how they were calculated

Our web collection on statistics for biologists contains articles on many of the points above.

Software and code

Policy information about availability of computer code

Data collection	Immunofluorescence: DeltaVision softWoRx 5.5<br>Mass spectrometry acquisition: Xcalibur 3.1 (Thermo Fisher Scientific), Paradigm Home v.2.0.4 R4 B22 (Bruker Daltonics), and Cycle Composer v.1.6.0 (CTC Analytics AG)<br>RT-qPCR: StepOnePlus Real-Time PCR System v2.2 (Applied Biosystems)<br>Cytokine assay: Bio-Plex Manager sofware version 5.0 (Bio-Rad)<br>Flow cytometry for EGFP-positive cells quantification: BD Accuri C6 Plus Software v.1.0.23.1 for BD Accuri C6 Plus Flow Cytometer (BD Biosciences)<br>Flow cytometry for cell sorting: BD FACSDiva Software v.6.1.3 for BD FACSAria III Flow Cytometer (BD Biosciences)
Data analysis	L1 ORF1p crystal structure: ChimeraX 1.2.5 for Windows (https://www.rbvi.ucsf.edu/chimerax/)<br>Mass spectrometry analyses: Proteome Discoverer 2.3, MASCOT 2.7.0 server, Database for Annotation, Visualization and Integrated Discovery 2021 Gene Ontology analysis (https://david.ncifcrf.gov/), Gene Set Enrichment Analysis (GSEA) 4.2.3 for Windows (https://www.gsea-msigdb.org/gsea)<br>Western blot quantification: ImageJ 1.52a for Windows (https://imagej.nih.gov/ij/)<br>RT-qPCR: StepOnePlus Real-Time PCR System v2.2 (Applied Biosystems)<br>Flow cytometry for EGFP-positive cells quantification: BD Accuri C6 Plus Software v.1.0.23.1 for BD Accuri C6 Plus Flow Cytometer (BD Biosciences)<br>Statistical analyses: ASTATSA 2016 (https://astatsa.com/), GraphPad Prism 9.0.0 for Windows

For manuscripts utilizing custom algorithms or software that are central to the research but not yet described in published literature, software must be made available to editors and reviewers. We strongly encourage code deposition in a community repository (e.g. GitHub). See the Nature Portfolio guidelines for submitting code & software for further information.

Data

Policy information about availability of data

All manuscripts must include a data availability statement. This statement should provide the following information, where applicable:

- Accession codes, unique identifiers, or web links for publicly available datasets
- A description of any restrictions on data availability
- For clinical datasets or third party data, please ensure that the statement adheres to our policy

The crystal structure images of ORF1p are based on the 2ykp pdb file (https://www.rcsb.org/structure/2ykp). Uniprot database (https://www.uniprot.org/help/uniprotkb) was used for protein identification from the mass spectrometry data and the Mascot Server 2.7.0 database (https://www.matrixscience.com) was used as the search engine. The mass spectrometry data will be available at jPOST repository (https://repository.jpostdb.org/) with the accession numbers (PXD032869 and PXD036759) or via ProteomeXchange with identifier PXD038851. Preranked GSEA analysis was performed using GSEA Molecular Signatures Database (MSigDB: https://www.gsea-msigdb.org/gsea/msigdb/), STRING database v11.5 (https://string-db.org/) was used for STRING analysis, and ISG screening was done using the interferome database v2.0 (www.interferome.org). Other data are included in this article and in the supplementary files. Source data including analyzed mass spectrometry data and uncropped western blot images are available in a separate file.

Human research participants

Policy information about studies involving human research participants and Sex and Gender in Research.

Reporting on sex and gender	No human research participant is involved in this study.
Population characteristics	No human research participant is involved in this study.
Recruitment	No human research participant is involved in this study.
Ethics oversight	No human research participant is involved in this study.

Note that full information on the approval of the study protocol must also be provided in the manuscript.

Field-specific reporting

Please select the one below that is the best fit for your research. If you are not sure, read the appropriate sections before making your selection.

☒ Life sciences ☐ Behavioural & social sciences ☐ Ecological, evolutionary & environmental sciences

For a reference copy of the document with all sections, see nature.com/documents/nr-reporting-summary-flat.pdf

Life sciences study design

All studies must disclose on these points even when the disclosure is negative.

Sample size	A sample size of at least three was chosen to ensure reproducibility and to allow statistical significance calculation following the general standards in the field. Notable references include Tunbak H. et al., Nature Communications, 2020; Tristán-Ramos P. et al., Nature Communications, 2020; Hwang S.-Y. et al, Nature Communications, 2021).
Data exclusions	Data were not excluded from the analyses.
Replication	All experiments were performed at least three times with appropriate positive and negative controls, unless stated otherwise. The number of replicates are indicated in each figure legend, respectively.
Randomization	Allocation of all samples into experimental groups was random and groups were treated similarly.
Blinding	The investigators who carried out the experiments were not blinded to the groups as all groups were treated similarly.

Reporting for specific materials, systems and methods

We require information from authors about some types of materials, experimental systems and methods used in many studies. Here, indicate whether each material, system or method listed is relevant to your study. If you are not sure if a list item applies to your research, read the appropriate section before selecting a response.

Materials & experimental systems

n/a	Involved in the study
☐	☒ Antibodies
☐	☒ Eukaryotic cell lines
☒	☐ Palaeontology and archaeology
☒	☐ Animals and other organisms
☒	☐ Clinical data
☒	☐ Dual use research of concern

Methods

n/a	Involved in the study
☒	☐ ChIP-seq
☐	☒ Flow cytometry
☒	☐ MRI-based neuroimaging

Antibodies

Antibodies used

Western Blot antibodies

Primary antibodies and dilutions (in parentheses):
Mouse monoclonal anti-FLAG M2 antibody (1/5000), (Sigma-Aldrich, F1804, 1.0 mg/mL, RRID: AB_262044)
Rabbit polyclonal anti-FLAG antibody (1/5000), (Sigma-Aldrich, F7425, ~0.8 mg/mL, RRID: AB_439687)
Mouse monoclonal anti-MYC antibody (1/5000), (Cell Signaling Technology, 9B11, RRID: AB_331783)
Rabbit polyclonal anti-PABPC1 antibody (1/5000), (Abcam, ab21060, 0.9 mg/mL, RRID: AB_777008)
Mouse monoclonal anti-GAPDH antibody (1/5000), (Millipore, MAB374, 1.0 mg/mL, RRID: AB_2107445)
Mouse monoclonal anti-Actin antibody (1/5000, after dilution to 0.2 times of the original concentration), (Millipore, MAB1501R, RRID: AB_2223041)
Rabbit polyclonal anti-T7-tag antibody (1/5000), (Cell Signaling Technology, D9E1X, RRID: AB_2798161)
Goat polyclonal anti-Luciferase antibody (1/2000), (Promega, G7451, 1.0 mg/mL, RRID: AB_430862)
Mouse monoclonal anti-ORF1p (4H1) antibody (1/2000), (Millipore, MABC1152, 0.5 mg/mL)
Mouse monoclonal anti-eIF3 p110 (B-6) antibody (1/5000), (Santa Cruz Biotechnology, sc-74507, 0.2 mg/mL, RRID: AB_1122487)
Secondary antibodies and dilutions (in parentheses):
Sheep polyclonal anti-mouse HRP-conjugated Whole antibody (1/5000), (GE Healthcare, NA931-1ML, RRID: AB_772210)
Goat polyclonal anti-rabbit HRP-conjugated Whole antibody (1/5000), (Cell Signaling Technology, 7074, RRID: AB_2099233)
Donkey polyclonal anti-rabbit HRP-conjugated Whole antibody (1/5000), (GE Healthcare, NA934-1ML, RRID: AB_772206)
Donkey polyclonal anti-goat HRP-conjugated Whole antibody (1/5000), (Santa Cruz Biotechnology, sc-2020, 0.4 mg/mL, RRID: AB_631728)

Immunofluorescence antibodies

Primary antibodies and dilutions (in parentheses):
Mouse monoclonal anti-FLAG M2 antibody (1/1000), (Sigma-Aldrich, F3165, 3.8 - 4.2 mg/mL, RRID: AB_259529)
Rabbit polyclonal anti-FLAG antibody (1/1000), (Sigma-Aldrich, F7425, ~0.8 mg/mL, RRID: AB_439687)
Mouse monoclonal anti-MYC antibody (1/1000), (Cell Signaling Technology, 9B11, RRID: AB_331783)
Secondary antibodies and dilutions (in parentheses):
Donkey anti-mouse polyclonal Alexa Fluor 488 IgG (H+L) (1/250), (Thermo Fisher Scientific, A-21202, 2.0 mg/mL, RRID: AB_141607)
Donkey anti-rabbit polyclonal Alexa Fluor 488 IgG (H+L) (1/250), (Thermo Fisher Scientific, A-21206, 2.0 mg/mL, RRID: AB_2535792)
Goat polyclonal anti-mouse Cy5 (1/250), (Jackson ImmunoResearch Labs, 115-175-146, RRID: AB_2338713)

Validation

Antibodies were validated by the manufacturers (anti-FLAG M2, anti-FLAG, anti-MYC, anti-PABPC1, anti-GAPDH, anti-Actin, anti-T7, anti-Luciferase, anti-ORF1p, anti-eIF3 p110, Sheep anti-mouse HRP, Goat anti-rabbit HRP, Donkey anti-rabbit HRP, Donkey anti-goat HRP for Western Blot; anti-MYC, anti-mouse Alexa Fluor 488, and anti-rabbit Alexa Fluor 488 for immunofluorescence).

Primary antibodies species reactivity validation provided by the manufacturers:
Mouse monoclonal anti-FLAG M2 antibody (Sigma-Aldrich, F1804, 1.0 mg/mL, RRID: AB_262044) - All
Mouse monoclonal anti-FLAG M2 antibody (Sigma-Aldrich, F3165, 3.8 - 4.2 mg/mL, RRID: AB_259529) - All
Rabbit polyclonal anti-FLAG antibody (Sigma-Aldrich, F7425, ~0.8 mg/mL, RRID: AB_439687) - All
Mouse monoclonal anti-MYC antibody (Cell Signaling Technology, 9B11, RRID: AB_331783) - All
Rabbit polyclonal anti-PABPC1 antibody (Abcam, ab21060, 0.9 mg/mL, RRID: AB_777008) - Mouse, Rat, Human. Predicted to react with Cow and Pig.
Mouse monoclonal anti-GAPDH antibody (Millipore, MAB374, 1.0 mg/mL, RRID: AB_2107445) - Canine, Human, Mouse, Rat, Rabbit, Fish, Feline, Pig
Mouse monoclonal anti-Actin antibody (Millipore, MAB1501R, RRID: AB_2223041) - All
Rabbit polyclonal anti-T7-tag antibody (Cell Signaling Technology, D9E1X, RRID: AB_2798161) - All
Goat polyclonal anti-Luciferase antibody (Promega, G7451, 1.0 mg/mL, RRID: AB_430862) - Specific for firefly luciferase (Photinus pyralis); antibody does not cross-react with sea pansy (Renilla reniformis) luciferase
Mouse monoclonal anti-ORF1p (4H1) antibody (Millipore, MABC1152, 0.5 mg/mL) - Human
Mouse monoclonal anti-eIF3 p110 (B-6) antibody (Santa Cruz Biotechnology, sc-74507, 0.2 mg/mL, RRID: AB_1122487) - Mouse, Rat, Human, Equine, Canine, Bovine

For western blot, we also verified the antibody by looking at the molecular weight and compare with appropriate negative controls. For immunofluorescence, we immunostained all cells similarly in each experiment and compared the transfected and non-transfected cells with epitope-tagged proteins to ensure antibodies specificity.

Eukaryotic cell lines

Policy information about cell lines and Sex and Gender in Research

Cell line source(s)	HEK293T cells were provided by ATCC, HeLa-JVM cells were provided by John V. Moran lab (University of Michigan), HeLa-HA from Astrid M. Roy-Engel lab (Tulane University), and U-2 OS from Roger Reddel lab (The University of Sydney).
Authentication	We confirm the identity of HeLa-JVM, HeLa-HA, U-2 OS, and HEK293T cells using STR-genotyping. In addition, we monitor the morphology and growth of these cell types based on prior experience.
Mycoplasma contamination	All cell lines were tested negative for mycoplasma contamination using a PCR-based method using the VenorGeM Classic Mycoplasma Detection Kit (Sigma-Aldrich).
Commonly misidentified lines (See ICLAC register)	No commonly misidentified cell lines was used in the study.

Flow Cytometry

Plots

Confirm that:

☒ The axis labels state the marker and fluorochrome used (e.g. CD4-FITC).

☒ The axis scales are clearly visible. Include numbers along axes only for bottom left plot of group (a 'group' is an analysis of identical markers).

☒ All plots are contour plots with outliers or pseudocolor plots.

☒ A numerical value for number of cells or percentage (with statistics) is provided.

Methodology

Sample preparation	Cells were trypsinized, resuspended in culture media, washed twice with cold 1x PBS, filtered, resuspended in cold 1x PBS (or 2% FBS in 1x PBS for cell sorting), and analysed using the flow cytometer.
Instrument	BD Accuri C6 Plus Flow Cytometer (BD Biosciences) for EGFP-positive cells quantification<br>BD FACSAria III Flow Cytometer (BD Biosciences) for cell sorting
Software	BD Accuri C6 Plus Software v.1.0.23.1 for BD Accuri C6 Plus Flow Cytometer (BD Biosciences)<br>BD FACSDiva Software v.6.1.3 for BD FACSAria III Flow Cytometer (BD Biosciences)
Cell population abundance	For EGFP-positive cells quantification, transfected cells were selected using antibiotics (blasticidin or puromycin) for 5-7 days. The FITC channel was used to count the EGFP positive cells out of 30,000 cells.<br>For cell sorting, the transfected cells were treated with G418 until all of the non-transfected controls are dead. The cells were transferred to a new 10-cm dish and grown until ~90% confluency before the sorting (single cell sorting). The PE channel was used to select 5000 dTomato positive cells.
Gating strategy	For all experiments, live cells were gated on by excluding debris, based on the forward and side scatter profiles of each cell line. A gate of EGFP or dTomato positive cells was made using the negative control sample and copied to all samples. Representative flow cytometry gating examples are shown in Supplementary Fig. 4a.

☒ Tick this box to confirm that a figure exemplifying the gating strategy is provided in the Supplementary Information.
